# Vaccinomics Approach to the Identification of Candidate Protective Antigens for the Control of Tick Vector Infestations and *Anaplasma phagocytophilum* Infection

**DOI:** 10.3389/fcimb.2017.00360

**Published:** 2017-08-09

**Authors:** Marinela Contreras, Pilar Alberdi, Isabel G. Fernández De Mera, Christoph Krull, Ard Nijhof, Margarita Villar, José De La Fuente

**Affiliations:** ^1^SaBio, Instituto de Investigación en Recursos Cinegéticos IREC-CSIC-UCLM-JCCM Ciudad Real, Spain; ^2^Institute for Parasitology and Tropical Veterinary Medicine, Freie Universität Berlin Berlin, Germany; ^3^Department of Veterinary Pathobiology, Center for Veterinary Health Sciences, Oklahoma State University Stillwater, OK, United States

**Keywords:** anaplasmosis, immunology, vaccine, tick, *Ixodes*, *Anaplasma phagocytophilum*

## Abstract

*Anaplasma phagocytophilum* is an emerging tick-borne pathogen causing human granulocytic anaplasmosis (HGA), tick-borne fever (TBF) in small ruminants, and other forms of anaplasmosis in different domestic and wild animals. The main vectors of this pathogen are *Ixodes* tick species, particularly *I. scapularis* in the United States and *I. ricinus* in Europe. One of the main limitations for the development of effective vaccines for the prevention and control of *A. phagocytophilum* infection and transmission is the identification of effective tick protective antigens. The objective of this study was to apply a vaccinomics approach to *I. scapularis*-*A. phagocytophilum* interactions for the identification and characterization of candidate tick protective antigens for the control of vector infestations and *A. phagocytophilum* infection. The vaccinomics pipeline included the use of quantitative transcriptomics and proteomics data from uninfected and *A. phagocytophilum*-infected *I. scapularis* ticks for the selection of candidate protective antigens based on the variation in tick mRNA and protein levels in response to infection, their putative biological function, and the effect of antibodies against these proteins on tick cell apoptosis and pathogen infection. The characterization of selected candidate tick protective antigens included the identification and characterization of *I. ricinus* homologs, functional characterization by different methodologies including RNA interference, immunofluorescence, gene expression profiling, and artificial tick feeding on rabbit antibodies against the recombinant antigens to select the candidates for vaccination trials. The vaccinomics pipeline developed in this study resulted in the identification of two candidate tick protective antigens that could be selected for future vaccination trials. The results showed that *I. scapularis* lipocalin (ISCW005600) and lectin pathway inhibitor (AAY66632) and *I. ricinus* homologs constitute candidate protective antigens for the control of vector infestations and *A. phagocytophilum* infection. Both antigens are involved in the tick evasion of host defense response and pathogen infection and transmission, but targeting different immune response pathways. The vaccinomics pipeline proposed here could be used to continue the identification and characterization of candidate tick protective antigens for the development of effective vaccines for the prevention and control of HGA, TBF, and other forms of anaplasmosis caused by *A. phagocytophilum*.

## Introduction

The intracellular bacterium, *Anaplasma phagocytophilum* (Rickettsiales: Anaplasmataceae) is an emerging tick-borne pathogen causing human granulocytic anaplasmosis (HGA), which has emerged as a tick-borne disease of humans in the United States, Europe and Asia, and tick-borne fever (TBF) in small ruminants, most notably in sheep in Europe (Gordon et al., 1932; Foggie, 1951; Dumler et al., 2001; Stuen et al., 2013; Bakken and Dumler, 2015; Dugat et al., 2015; Severo et al., 2015). Clinical presentation of *A. phagocytophilum* infection has been also documented in goats, cattle, horses, dogs, cats, roe deer, and reindeer (Severo et al., 2015). The main vectors of this pathogen are *Ixodes* tick species, particularly *I. scapularis* in the United States and *I. ricinus* in Europe (Stuen et al., 2013; Bakken and Dumler, 2015).

Despite the burden that *A. phagocytophilum* represents for humans and animals, vaccines are not available for prevention and control of pathogen infection and transmission (Dumler et al., 2001; Stuen et al., 2013, 2015; Bakken and Dumler, 2015; Severo et al., 2015; Contreras et al., 2017). One of the main limitations for the development of effective vaccines for the prevention and control of *A. phagocytophilum* infection and transmission is the identification of effective tick protective antigens. Recently, different approaches have been developed for the identification and characterization of candidate tick protective antigens (de la Fuente and Contreras, 2015; de la Fuente et al., 2016a). Vaccinomics is one of the approaches that have been used by our group for the identification of tick-derived and pathogen-derived protective antigens (de la Fuente and Merino, 2013; Merino et al., 2013; Antunes et al., 2014; de la Fuente and Contreras, 2015; Contreras et al., 2016, 2017; de la Fuente et al., 2016a; Villar et al., 2017). Vaccinomics is a holistic approach based on the use of genome-scale or omics technologies integrated in a systems biology approach to characterize tick-host-pathogen interactions for the development of next-generation vaccines (de la Fuente and Merino, 2013; Contreras et al., 2016; de la Fuente et al., 2016a; Villar et al., 2017). In this translational approach, basic biological information on tick-host-pathogen interactions translates into the identification and subsequent evaluation of new candidate protective antigens (de la Fuente and Merino, 2013; de la Fuente et al., 2016a; Villar et al., 2017).

The sequence, assembly and annotation of the *I. scapularis* genome were recently released (Gulia-Nuss et al., 2016), and various genomics, transcriptomics and proteomics studies in *I. ricinus* suggest that these tick species are genetically closely related (Schwarz et al., 2013, 2014; Genomic Resources Development Consortium et al., 2014; Cramaro et al., 2015; Kotsyfakis et al., 2015; Weisheit et al., 2015; Chmelař et al., 2016). These results open new opportunities for research on tick-host-pathogen interactions and the possibility of identifying tick protective antigens for both *I. scapularis* and I. *ricinus* major vectors of *A. phagocytophilum* (de la Fuente et al., 2016b).

Recently, transcriptomics, proteomics and metabolomics datasets have been integrated and used for the characterization of *I. scapularis*-*A. phagocytophilum* molecular interactions (Ayllón et al., 2015; Villar et al., 2015a,b, 2016; Cabezas-Cruz et al., 2016, 2017a,b; de la Fuente et al., 2016c, 2017; Gulia-Nuss et al., 2016; Shaw et al., 2017). Herein, a vaccinomics pipeline was developed based on quantitative transcriptomics and proteomics data from uninfected and *A. phagocytophilum*-infected *I. scapularis* nymphs, adult female midguts and salivary glands, and ISE6 cells (Ayllón et al., 2015; Villar et al., 2015a). The vaccinomics pipeline was then used for the identification of candidate protective antigens for the control of vector infestations and pathogen infection. The results showed that *I. scapularis* ISCW005600 and AAY66632 and *I. ricinus* homologs constitute candidate protective antigens for the control of vector infestations and *A. phagocytophilum* infection.

## Materials and methods

### Ticks and cultured tick cells

*Ixodes scapularis* ticks were obtained from the laboratory colony maintained at the Oklahoma State University Tick Rearing Facility. Nymphs and adult female *I. scapularis* were infected with *A. phagocytophilum* by feeding on a sheep inoculated intravenously with approximately 1 × 10^7^ *A. phagocytophilum* (NY18 isolate)-infected HL-60 human cells (90–100% infected cells) (Kocan et al., 2012; Ayllón et al., 2015). Animals were housed and experiments conducted with the approval and supervision of the OSU Institutional Animal Care and Use Committee (Animal Care and Use Protocol, ACUP No. VM1026). *I. ricinus* ticks were obtained from the laboratory colony maintained at the Freie Universität Berlin. Larvae and nymphs were fed on mice and adults on rabbits. The *I. scapularis* embryo-derived tick cell line ISE6, provided by Ulrike Munderloh, University of Minnesota, USA, was cultured in L-15B300 medium as described previously (Kurtti et al., 1996; Munderloh et al., 1999; Villar et al., 2015a). IRE/CTVM20 embryo-derived tick cells, provided by the Tick Cell Biobank, were maintained as described previously (Bell-Sakyi et al., 2007; Alberdi et al., 2015). Tick cells were first inoculated with *A. phagocytophilum* (human NY18 isolate; Asanovich et al., 1997)-infected HL-60 cells and maintained according to Munderloh et al. (1999). Uninfected and infected cultures (*N* = 4 independent cultures with approximately 10^7^ cells each) were sampled at 7 days post-infection (dpi) (75% infected cells). The percentage of cells infected with *A. phagocytophilum* was calculated by examining at least 200 cells using a 100x oil immersion objective.

### Transcriptomics and proteomics datasets

The quantitative transcriptomics and proteomics data for uninfected and *A. phagocytophilum*-infected *I. scapularis* nymphs, adult female midguts and salivary glands, and ISE6 cells were obtained from previously published results (Ayllón et al., 2015; Villar et al., 2015a) and deposited at the Dryad repository database, NCBI's Gene Expression Omnibus database and ProteomeXchange Consortium via the PRIDE partner repository with the dataset identifier PXD002181 and doi: 10.6019/PXD002181.

### Sequence analysis

To find the *I. ricinus* homologs, selected *I. scapularis* sequences were blasted against the *I. ricinus* database using the Blastp tool from BLAST (Altschul et al., 1990; Madden et al., 1996), and the sequences with the lowest *E*-value were selected. Gene ontology (GO) analysis for biological process (BP) was done with Blast2GO software (version 3.0; http://www.blast2go.com) (Villar et al., 2014).

### Production of recombinant proteins

The coding sequences for *I. scapularis* candidate protective antigens were amplified from synthetic genes optimized for codon usage in *Escherichia coli* (Genscript Corporation, Piscataway, NJ, USA) using sequence-specific primers (Table 1). The amplified DNA fragments were cloned into the expression vector pET101 and expressed in *E. coli* strain BL21 using the Champion pET101 Directional TOPO Expression kit (Carlsbad, CA, USA). Recombinant proteins were fused to Histidine tags for purification by affinity to Ni (Merino et al., 2013; Moreno-Cid et al., 2013). Transformed *E. coli* strains were induced with IPTG for 4.5 h to produce recombinant proteins, which were purified to >85% of total cell proteins by Ni affinity chromatography (Genscript Corporation) as previously described (Merino et al., 2013; Moreno-Cid et al., 2013) using 1 ml HisTrap FF columns mounted on an AKTA-FPLC system (GE Healthcare, Piscataway, NJ, USA) in the presence of 7 M urea lysis buffer. The purified antigens were refolded by dialysis against 1,000 volumes of PBS, pH 7.4 (137 mM NaCl, 2.7 mM KCl, 10 mM Na_2_HPO_4_, 1.8 mM KH_2_PO_4_) for 12 h at 4°C.

**Table 1:** Oligonucleotide primers used in this study for cloning, RNAi and RT-PCR.

**GenBank accession No**.	**Oligonucleotide sequence (5′-3′ for forward and reverse primers)**
**CLONING**	
ISCW024685	CACCATGAAAAGCAGCGCACTGCTG
	GCGTTTACCACGAACGCACC
ISCW024295	CACCATGCCGAAACAAGGCGAAAC
	TCCAGAGTCACCACACAAAACG
ISCW022212	CACCATGTGGGGTCAGATTGCGCT
	ACAGATGAATTTTTTCAGGC
ISCW020900	CACCATGAACAAAGCGATCTTCAT
	CACTTCACCGAAAAAGCCGC
ISCW000326	CACCATGCCGGCGTCAATGAAAAG
	CAGAGAACCCAGATTCGGAA
ISCW008146	CACCATGGATTTTGATGACCTGTT
	GAAGCTCAGGGTGTTCTGTT
ISCW008641	CACCATGCAACGTGACATTTTTAG
	CCAACAGCCCGGCTGCGATT
ISCW024499	CACCATGTGCCTGGTGTTTGCAAC
	GCGCAGAAAGGAACTCGTAC
ISCW005600	CACCATGATTCGTCAGGTTCGCGA
	CGAACCTGAGATCGATGAGG
ISCW013709	CACCATGTTTCGTACCAGCTCTGG
	CACAATATAATCCGGTGCAC
ISCW017117	CACCATGCTGAGTGTGCTGCTGGG
	CGTGGTGGCGTCCGGCGGCG
ISCW013574	CACCATGTATCAGCTGCGCGATTT
	GCAACGGGATTTGCGAACAC
ISCW018900	CACCATGGGCCCGTTTATTGGTCT
	GCCGATAATGCGACCGATAA
ISCW023907	CACCATGCCGGTCAATCGCCTGAT
	AACTTTACGAAAGAAAAACA
ISCW024682	CACCATGATTCATGAACCGGTGAT
	ATACGGACAGTACAGTTTGCA
ISCW015453	CACCATGATGAAAAGCCCGCTGTTTAT
	ACCGAAAAAGCCGTGGCCGA
ISCW021670	CACCATGTGGGAACTGCATGCCGA
	CTCCTGGGTAATATTACGCGT
ISCW017271	CACCATGTGCAGCGATTCTAAACC
	CGGCAGATAGGAACCGTGCG
AAY66632	CACCATGGGCCTGACCGGTACCAC
	GTTGTCTTTGGTTTTCTTGG
**RNAi AND RT-PCR**	
ISCW005600	TCCCCTTCTCAAAGGAGGAT^*^
	ATCCACAGGCGGATATGAAG^*^
AAY66632	ACCCGTTCATGGGACAAATA^*^
	TTCTTGGGCTTCTCAGTTGG^*^
DQ066214 (*rpS4*)	GGTGAAGAAGATTGTCAAGCAGAG
	TGAAGCCAGCAGGGTAGTTTG

* *The same oligonucleotide primers were used to determine gene expression levels by RT-PCR and for the generation of dsRNA for RNAi. To produce dsRNA, the T7 promoter sequence 5′-GAATTAATACGACTCACTATAGGGAGA-3′was added to the 5′-end of each primer*.

### Production of rabbit polyclonal IgG antibodies

For each recombinant tick protein and total ISE6 tick cell proteins, two New Zealand white rabbits (*Oryctulagus cuniculus*) were subcutaneously injected at weeks 0, 4, and 6 with 50 μg protein in 0.4 ml Montanide ISA 50 V adjuvant (Seppic, Paris, France). Blood was collected before injection and 2 weeks after the last immunization to prepare pre-immune and immune sera, respectively. Serum aliquots were kept at 4°C for immediate use or at −20°C for long-term storage. The IgG were purified from serum samples using the Montage antibody purification kit and spin columns with PROSEP-A media (Millipore, Billerica, MA, USA) following the manufacturer's recommendations.

### Western blot analysis

Ten micrograms of each recombinant protein or 20 μg total proteins from ISE6 tick cells were loaded onto a 12% SDS-polyacrylamide pre-cast gel (Life Science, Hercules, CA, USA) and transferred to a nitrocellulose membrane. The membrane was blocked with 5% bovine serum albumin (BSA) (Sigma-Aldrich, St. Louis, MI, USA) for 2 h at room temperature (RT), and washed four times with TBS (50 mM Tris-Cl, pH 7.5, 150 mM NaCl, 0.5% Tween 20). Purified rabbit IgG were used at a 1:500 dilution in TBS, and the membrane was incubated overnight at 4°C and washed four times with TBS. The membrane was then incubated with an anti-rabbit IgG-horseradish peroxidase (HRP) conjugate (Sigma-Aldrich) diluted 1:1,000 in TBS with 3% BSA. The membrane was washed five times with TBS and finally developed with TMB (3,3′, 5,5′- tetramethylbenzidine) stabilized substrate for HRP (Promega, Madrid, Spain) according to the manufacturer recommendations.

### Immunofluorescence assay (IFA) in adult female ticks

Adult *I. scapularis* females were infected with *A. phagocytophilum* (NY18) as described above. Female ticks were removed from the sheep 10 days after infestation, held in the humidity chamber for 4 days and fixed with 4% paraformaldehyde in 0.2 M sodium cacodylate buffer, dehydrated in a graded series of ethanol and embedded in paraffin (Ayllón et al., 2015). Sections (4 μm) were prepared and mounted on glass slides. The paraffin was removed from the sections with xylene and the sections were hydrated by successive 2 min washes with a graded series of 100, 95, 80, 75, and 50% ethanol. The slides were treated with Proteinase K (Dako, Barcelona, Spain) for 7 min, washed with PBS and incubated with 3% BSA (Sigma-Aldrich) in PBS for 1 h at RT. The slides were then incubated for 14 h at 4°C with primary rabbit IgG antibodies diluted 1:100 in 3% BSA/PBS and, after 3 washes in PBS, developed for 1 h with goat-anti-rabbit IgG conjugated with phycoerythrin (PE) (Sigma-Aldrich) (diluted 1:50 in 3% BSA/PBS). The slides were washed twice with PBS and mounted in ProLong Antifade with DAPI reagent (Molecular Probes, Eugene, OR, USA). The sections were examined using a Zeiss LSM 800 laser scanning confocal microscope (Carl Zeiss, Oberkochen, Germany). Sections of uninfected ticks and IgG from pre-immune and anti-ISE6 sera were used as controls.

### Antibody inhibition assay

The inhibitory effect of rabbit IgG antibodies on *A. phagocytophilum* (NY18) was conducted as described previously (Villar et al., 2015b). ISE6 and IRE/CTVM20 tick cells were pooled and used to seed 24-well plates for each assay. Each well received 1 × 10^6^ cells in L-15B300 (ISE6) or L-15/L-15B (IRE/CTVM20) medium 24 h prior to inoculation with *A. phagocytophilum*. Infected cultures for inoculum were harvested when infection reached 80% and host cells were mechanically disrupted with a syringe and 26-gauge needle. Purified IgG (100 μg/ml) were added to the culture media and incubated with the cells for 48 h. Then, the medium with antibodies was removed and the *A. phagocytophilum* inoculum (100 μl) was added to the cell monolayers and incubated at 31°C for 60 min. The inoculum was removed from the wells and cell monolayers washed three times with PBS. Complete medium (1 ml) was added to each well and the plates were incubated at 31°C. The control included inoculum incubated with rabbit pre-immune and anti-ISE6 IgG. Four replicates were done for each treatment. After 72 h, cells from all wells were harvested and processed for *A. phagocytophilum* detection by real-time PCR after DNA extraction. Results were compared between treatments by the Student's *t*-test with unequal variance (*P* = 0.05; *N* = 4 biological replicates).

### Flow cytometry of tick cells incubated with rabbit IgG antibodies

Approximately 5 × 10^5^–1 × 10^6^ of *A. phagocytophilum*-infected ISE6 and IRE/CTVM20 tick cells were collected after incubation with rabbit IgG. Purified IgG (2.2–2.4 mg/ml) were mixed with *A. phagocytophilum* and incubated with tick cells as described above in the antibody inhibition assay. Apoptosis was measured by flow cytometry using the Annexin V-fluorescein isothiocyanate (FITC) apoptosis detection kit (Immunostep, Salamanca, Spain) following the manufacturer's protocols. The technique detects changes in phospholipid symmetry analyzed by measuring Annexin V (labeled with FITC) binding to phosphatidylserine, which is exposed in the external surface of the cell membrane in apoptotic cells. Cells were stained simultaneously with the non-vital dye propidium iodide (PI) allowing the discrimination of intact cells (Annexin V-FITC negative, PI negative) and early apoptotic cells (Annexin V-FITC positive, PI negative). All samples were analyzed on a FAC-Scalibur flow cytometer equipped with CellQuest Pro software (BD Biosciences, Madrid, Spain). The viable cell population was gated according to forward-scatter and side-scatter parameters. The percentage of apoptotic cells was determined by flow cytometry after Annexin V-FITC and PI labeling and compared between treated and untreated uninfected cells by Student's *t*-test with unequal variance (*P* = 0.05; *N* = 4 biological replicates).

### RNA interference (RNAi) for gene knockdown in tick cells

RNAi was used to characterize the effect of gene knockdown on tick cell pathogen infection. Oligonucleotide primers homologous to selected *I. scapularis* ISCW005600 and AAY66632 genes containing T7 promoters (Table 1) were used for *in vitro* transcription and synthesis of dsRNA as described previously (Ayllón et al., 2013), using the Access RT-PCR system (Promega, Madison, WI, USA) and the Megascript RNAi kit (Ambion, Austin, TX, USA). The unrelated *Rs86* dsRNA was synthesized using the same methods described previously and used as negative control (Ayllón et al., 2013). The dsRNA was purified and quantified by spectrophotometry. RNAi experiments were conducted in cell cultures by incubating ISE6 tick cells with 10 μl dsRNA (5 × 10^10^–5 × 10^11^ molecules/μl) and 90 μl L15B300 medium in 24-well plates using 5 wells per treatment (Ayllón et al., 2013). Control cells were incubated with the unrelated *Rs86* dsRNA. After 48 h of dsRNA exposure, tick cells were infected with cell-free *A. phagocytophilum* (NY18) obtained from approximately 5 × 10^6^ infected HL-60 cells (90–100% infected cells) (Thomas and Fikrig, 2007) and resuspended in culture medium to use 1 ml/well. Cells were incubated for an additional 72 h, harvested and used for DNA and RNA extraction. RNA was used to analyze gene knockdown by real-time RT-PCR with respect to *Rs86* control. DNA was used to quantify the *A. phagocytophilum* infection levels by real-time PCR.

### Determination of *A. phagocytophilum* infection by real-time PCR

*A. phagocytophilum* DNA levels were characterized by *major surface protein 4* (*msp4*) real-time PCR normalized against tick *ribosomal protein S4* (*rpS4*) as described previously (Ayllón et al., 2015). Normalized Ct-values were compared between untreated and treated cells by Student's *t*-test with unequal variance (*P* = 0.05; *N* = 4 biological replicates).

### Determination of tick mRNA levels by real-time RT-PCR

Total RNA was extracted from ISE6 tick cell cultures using TriReagent (Sigma-Aldrich) following manufacturer's recommendations. The expression of selected *I. scapularis* ISCW005600 and AAY66632 genes was characterized using total RNA extracted from infected and uninfected ISE6 tick cells. Real-time RT-PCR was performed on RNA samples using gene-specific oligonucleotide primers (Table 1) and the Kapa SYBR Fast One-Step qRT-PCR Kit (Kapa Biosystems, Wilmington, MA, USA) and the Rotor-Gene Real-Time PCR Detection System (Qiagen, Madrid, Spain). A dissociation curve was run at the end of the reaction to ensure that only one amplicon was formed and that the amplicons denatured consistently at the same temperature range for every sample. The mRNA levels were normalized against tick *rpS4* using the genNorm method (Delta-Delta-Ct, ddCT) as described previously (Ayllón et al., 2015). Normalized Ct-values were compared between infected and uninfected tick cells by Student's *t*-test with unequal variance (*P* = 0.05; *N* = 4 biological replicates).

### Artificial tick feeding

Artificial tick feeding was conducted as previously described for *Dermacentor reticulatus* (Krull et al., 2017). Briefly, 17–19 female and 3 male *I. ricinus* ticks were placed on each feeding unit. The feeding unit was subsequently closed by the insertion of a pierced plastic lid (PE-LD Stopfen 26 mm, Brimon Laborbedarf, Hamburg, Germany) wrapped in gauze fabric into the feeding unit, leaving approximately one cm between the silicone membrane and lid. The feeding unit was then hung into a glass beaker (50 ml, Simax, Czech Republic) containing the bovine blood using a rubber ring with an inner diameter of 32 mm (Lux, Wermelskirchen, Germany). Blood was supplemented with ATP and gentamycin (Krull et al., 2017), and 5 ml blood per feeding unit was pipetted into a sterile beaker and preheated to 37°C on a hot plate. The blood was changed twice daily at 12 ± 2 h intervals. During each blood change, the outside of the feeding unit and underside of the silicone membrane were rinsed with sterile 0.9% NaCl solution, pre-heated to body temperature. The number of attached, dead and fed ticks was counted after which the feeding unit was transferred to a new sterile beaker with fresh blood. Males stayed inside the feeding unit until the end of the experiment, to provide them with sufficient opportunity and time to fertilize any females present. Feeding units were placed in an incubator (ICH 256C, Memmert GmbH, Schwabach, Germany), where the blood was maintained at a constant temperature of 37°C using a heating plate (Hot Plate 062, Labotect, Göttingen, Germany). Environmental conditions were set at 20°C, 80% relative humidity, 5% CO_2_ and 15 h light/9 h dark. Once ticks were partially engorged, the feeding units were transferred to a six-well plate (Sarstedt, Nümbrecht, Germany) and ticks were fed for 36 h with 3 ml blood supplemented with 1 mg/ml of pre-immune or antigen-specific purified IgG. Dead and detached engorged ticks were removed, and engorged females that detached were weighed and stored individually in 2 ml Eppendorf tubes with pierced lids, which were kept in desiccators with approximately 90% relative humidity at RT. Ticks were assessed for egg mass 8 weeks post-feeding. The number of dead/fed ticks, ticks and eggs weight, and ticks with or without oviposition were compared between groups by a Fisher's exact test (*P* = 0.05; http://www.socscistatistics.com/tests/fisher/Default2.aspx).

## Results and discussion

### Selection of candidate tick protective antigens

**Figure 1:**
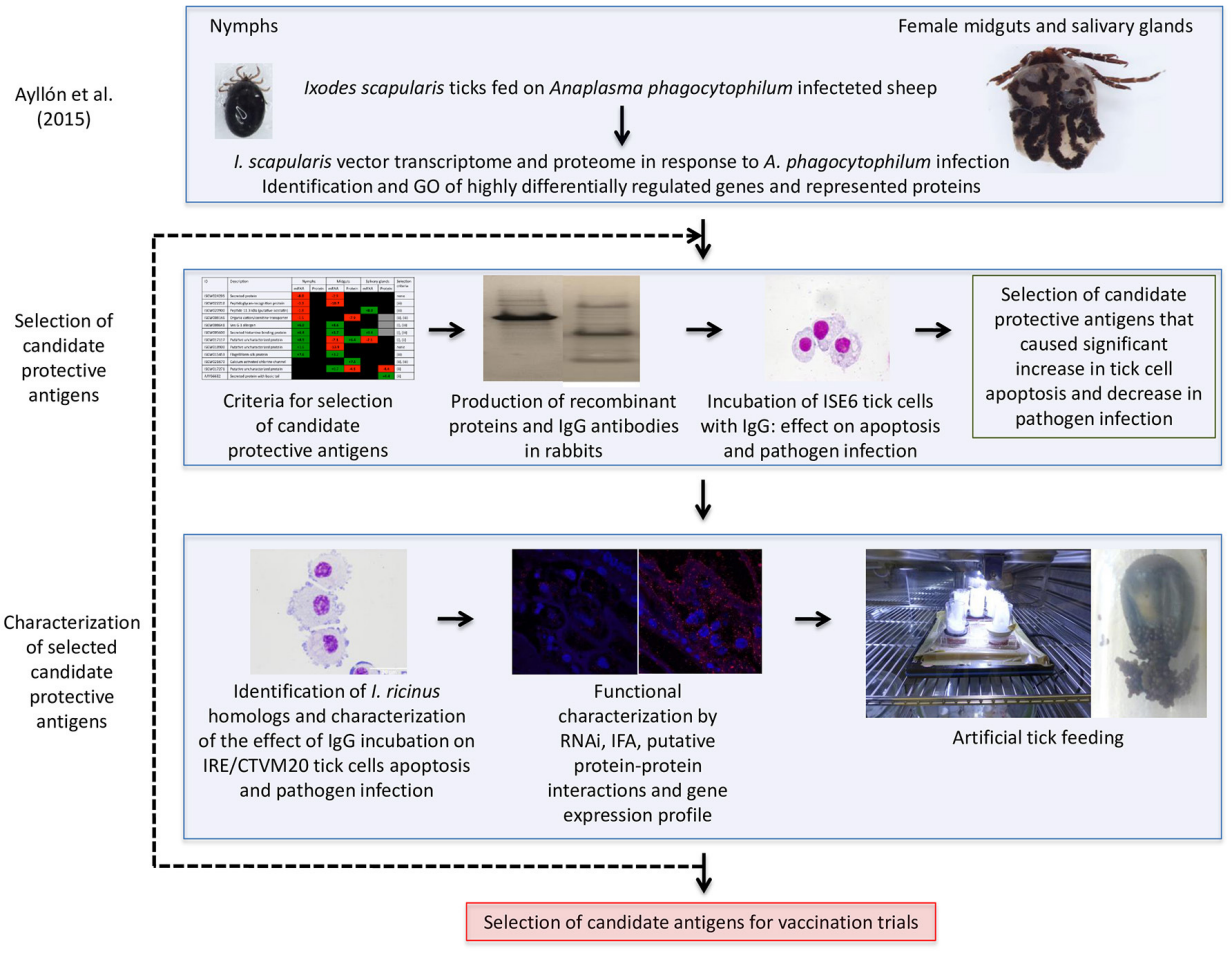
Vaccinomics pipeline for the selection and characterization of candidate tick protective antigens for the control of vector infestations and pathogen infection. The quantitative transcriptomics and proteomics data for uninfected and *A. phagocytophilum*-infected *I. scapularis* nymphs, adult female midguts and salivary glands were obtained from previously published results (Ayllón et al., 2015). The selection of candidate tick protective antigens included criteria based on the variation in tick mRNA and protein levels in response to infection, their putative biological function, and the effect of antibodies against these proteins on tick cell apoptosis and pathogen infection. The characterization of selected candidate protective antigens included the identification and characterization of *I. ricinus* homologs, functional analyses by different methodologies, and artificial tick feeding on rabbit antibodies against the recombinant antigens to select the candidates for vaccination trials.

A vaccinomics pipeline was developed for the selection and characterization of candidate tick protective antigens for the control of vector infestations and pathogen infection (Figure 1). The vaccinomics pipeline included the use of quantitative transcriptomics and proteomics data from uninfected and *A. phagocytophilum*-infected *I. scapularis* ticks (Ayllón et al., 2015) for the selection of candidate protective antigens based on the variation in tick mRNA and protein levels in response to infection, their putative biological function, and the effect of antibodies against these proteins on tick cell apoptosis and pathogen infection (Figure 1). The characterization of selected candidate tick protective antigens included the identification and characterization of *I. ricinus* homologs, functional characterization by different methodologies including RNAi, IFA, gene expression profile, and artificial tick feeding on rabbit antibodies against the recombinant antigens to select the candidates for vaccination trials (Figure 1). This process could be repeated as many times as needed to cover all potential candidate antigens or until the desired number of candidate antigens for vaccination trials is reached (Figure 1). The vaccinomics pipeline included some of the algorithms previously proposed (de la Fuente and Merino, 2013; Contreras et al., 2016) and validated (Merino et al., 2013; Antunes et al., 2014) for the selection and characterization of candidate protective antigens, but for the first time it was applied to integrated transcriptomics and proteomics data of tick-pathogen interactions.

**Figure 2:**
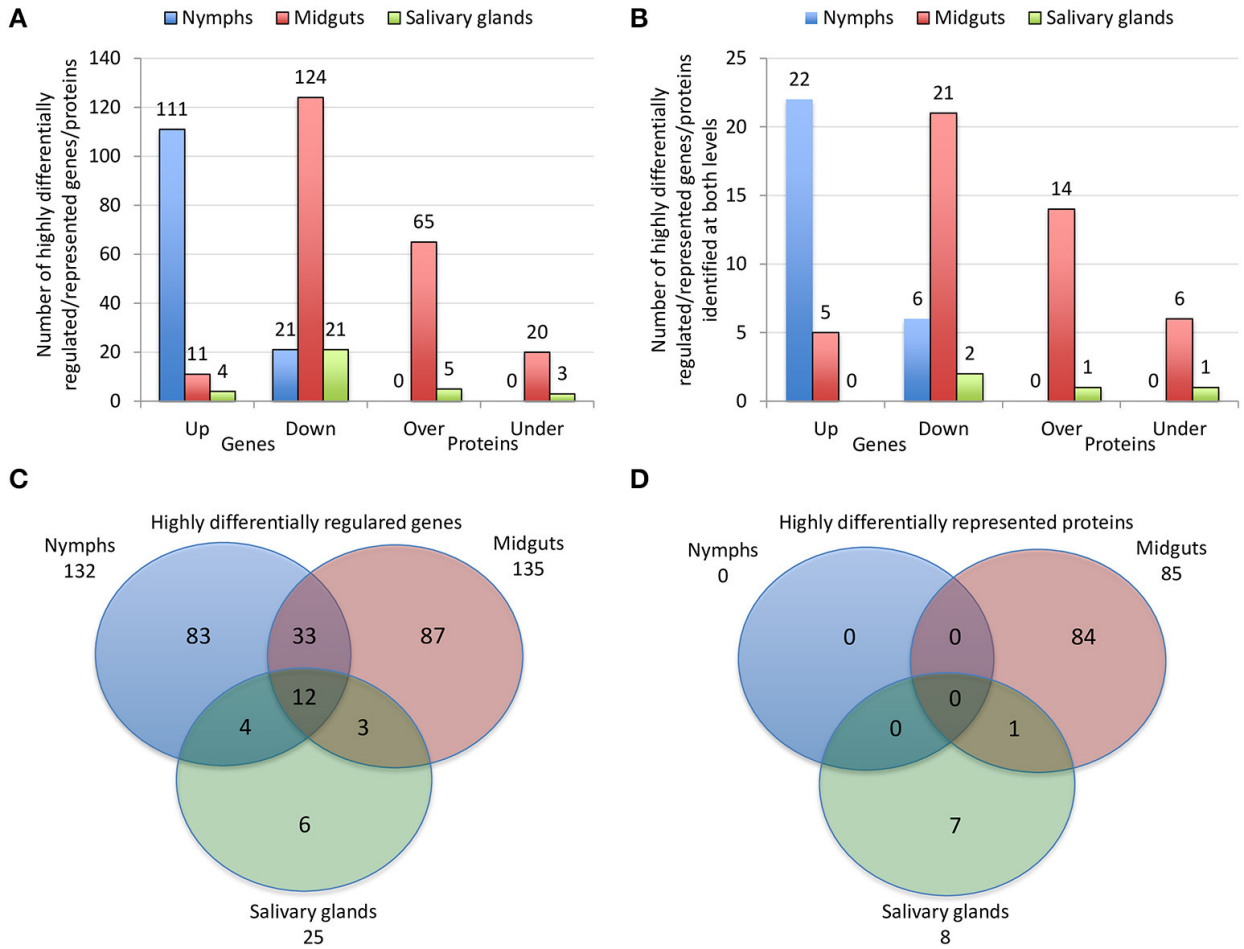
Effect of *A. phagocytophilum* infection on *I. scapularis* highly differentially regulated genes and represented proteins. **(A)** Number of tick highly differentially regulated genes and differentially represented proteins that were up-regulated (Up), down-regulated (Down), over-represented (Over) and under-represented (Under) in response to pathogen infection. **(B)** Number of tick highly differentially regulated genes and differentially represented proteins that were up-regulated (Up), down-regulated (Down), over-represented (Over) and under-represented (Under) in response to pathogen infection, and identified at both mRNA and protein levels. **(C)** Venn diagram with highly differentially regulated genes that were identified in tick nymphs, midguts and salivary glands. **(D)** Venn diagram with highly differentially represented proteins that were identified in tick nymphs, midguts and salivary glands. The highly differentially regulated genes were selected as those with more than 50-fold (log2 normalized fold change > 5.64) difference between infected and uninfected tick samples (*P* < 0.00003). The highly differentially represented proteins were selected as those with more than 15-fold (log2 normalized fold change > 3.90) change between infected and uninfected tick samples (*P* < 0.00003). The quantitative transcriptomics and proteomics data for uninfected and *A. phagocytophilum*-infected *I. scapularis* nymphs, adult female midguts and salivary glands were obtained from previously published results (Ayllón et al., 2015).

**Figure 3:**
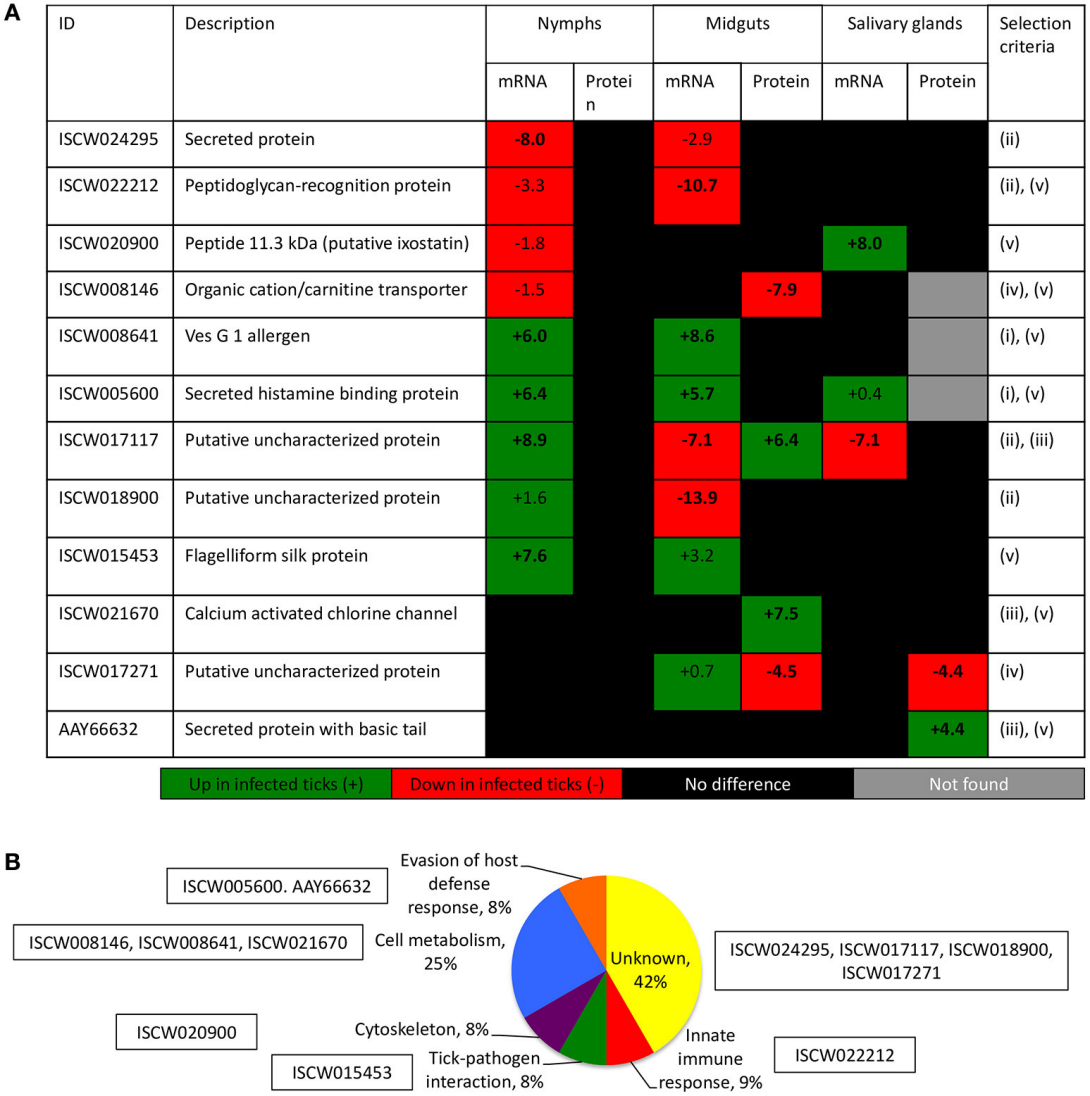
Criteria for the selection of candidate tick protective antigens. **(A)** Annotation and criteria used for the selection of candidate tick protective antigens. The log2 normalized fold change between infected and uninfected tick samples (*P* < 0.00003) is shown. Bold numbers indicate highly differentially regulated genes (log2 normalized fold change > 5.64) and highly differentially represented proteins (log2 normalized fold change > 3.90) between infected and uninfected tick samples. The selection criteria were (i) highly differentially up-regulated genes in at least two samples, (ii) highly down-regulated genes in at least one sample, (iii) highly differentially over-represented proteins and identified in the *I. scapularis* proteome, (iv) highly differentially under-represented proteins and identified in the *I. scapularis* proteome, and/or (v) putative BP in tick-pathogen and tick-host interactions. **(B)** The GO for BP of selected candidate protective antigens was done with Blast2GO software (version 3.0; http://www.blast2go.com).

The characterization of tick-pathogen molecular interactions was based on the previous work by Ayllón et al. (2015) of the *I. scapularis* transcriptome and proteome in response to *A. phagocytophilum* infection in nymphs and female midguts and salivary glands. The highly differentially regulated genes were selected as those with more than 50-fold (log2 normalized fold change >5.64) difference between infected and uninfected tick samples (*P* < 0.00003) (Figure 2A). The highly differentially represented proteins were selected as those with more than 15-fold (log2 normalized fold change >3.90) change between infected and uninfected tick samples (*P* < 0.00003) (Figure 2A). Of the highly differentially regulated/represented genes/proteins, between 0 and 50% were identified at both mRNA and protein levels in the different samples (Figure 2B). The analysis of highly differentially expressed/represented genes/proteins in response to *A. phagocytophilum* infection evidenced tissue-specific differences in response to infection (Ayllón et al., 2015), which were taken into consideration for the selection of candidate protective antigens (Figures 2A–D). The candidate protective antigens were selected by using the criteria (i) highly differentially up-regulated genes in at least two samples, (ii) highly down-regulated genes in at least one sample, (iii) highly differentially over-represented proteins and identified in the *I. scapularis* proteome, (iv) highly differentially under-represented proteins and identified in the *I. scapularis* proteome, and/or (v) putative BP in tick-pathogen and tick-host interactions (Figure 3A). The rationale behind the selection criteria for candidate protective antigens was based on their putative relevance in (i, iii) tick response to infection (de la Fuente et al., 2016c,d; 2017), (ii, iv) manipulated by *A. phagocytophilum* to decrease tick protective mechanisms and increase infection (de la Fuente et al., 2016c,d, 2017), and (v) tick-pathogen and tick-host interactions (Figure 3B).

**Figure 4:**
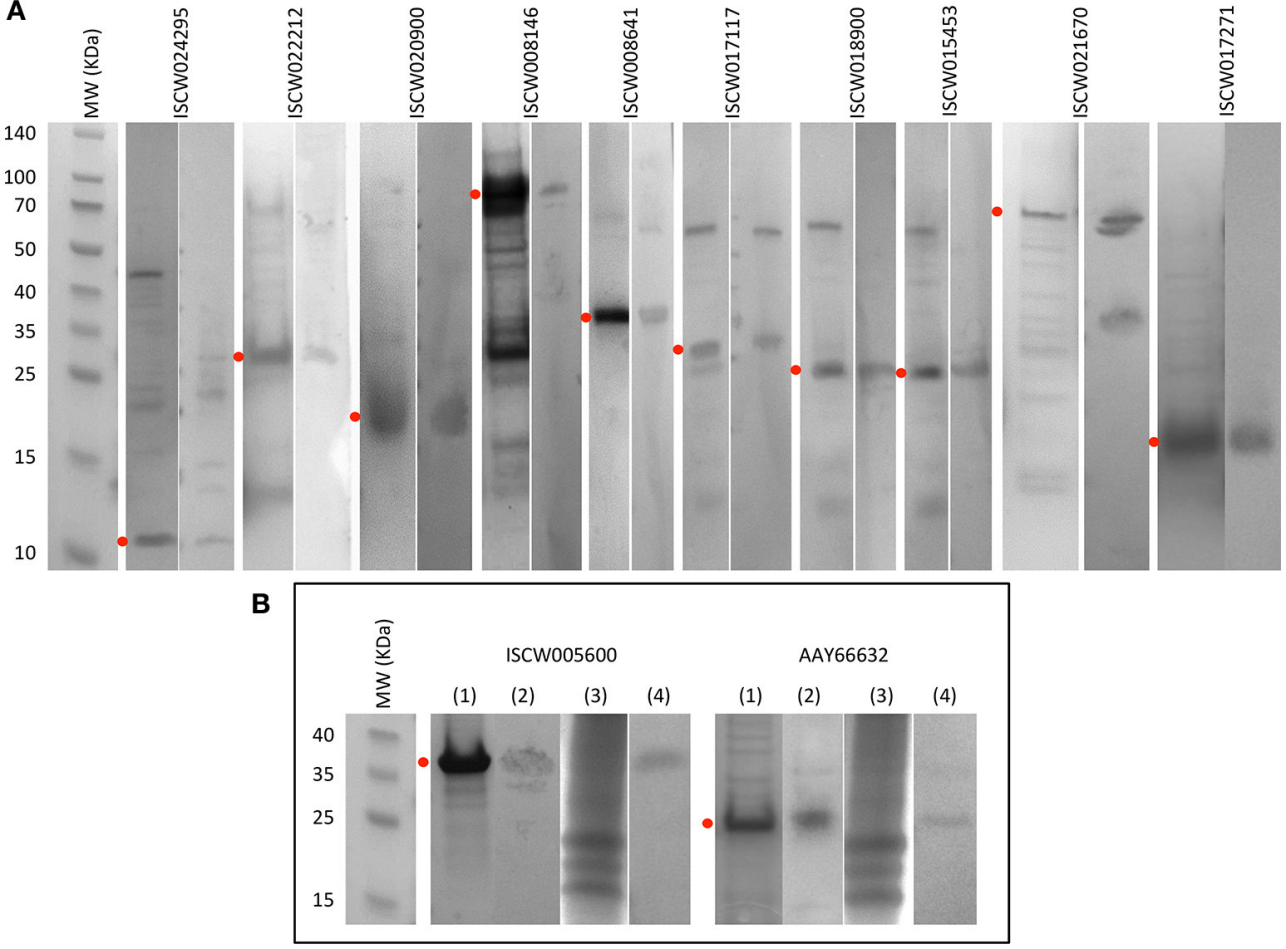
Production of recombinant proteins and rabbit IgG antibodies. **(A)** SDS-PAGE (left panel) and Western blot analysis (right panel) of selected candidate tick protective recombinant antigens. **(B)** Antigens selected for further characterization. (1) SDS-PAGE of recombinant antigens. (2) Western blot analysis of recombinant antigens. (3) SDS-PAGE of total proteins from ISE6 tick cells. (4) Western blot analysis of total proteins from ISE6 tick cells. Western blots were performed with IgG antibodies from rabbits immunized with recombinant antigens. Red dots denote the position of the recombinant antigen. Other protein bands in some of the samples correspond to *E. coli* contamination proteins, and aggregation or degradation products of the recombinant antigens.

**Figure 5:**
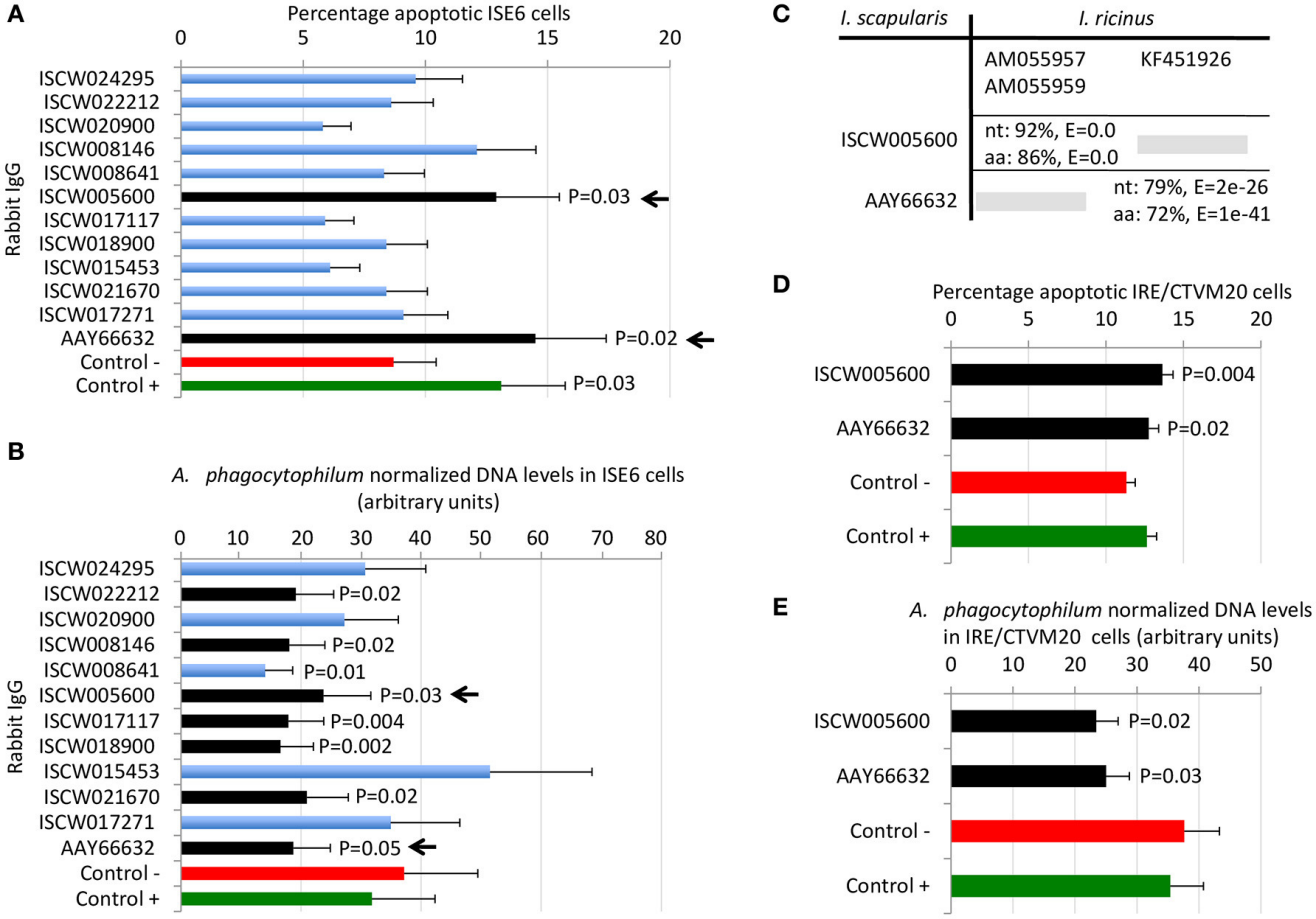
Selection of candidate tick protective antigens. Purified rabbit IgG against recombinant candidate tick protective antigens were incubated with *I. scapularis* ISE6 and *I. ricinus* IRE/CTVM20 tick cells before infection with *A. phagocytophilum*. **(A)** Characterization of the effect of rabbit IgG antibodies on ISE6 tick cells apoptosis. **(B)** Characterization of the effect of rabbit IgG antibodies on pathogen infection of ISE6 tick cells. **(C)** Sequence identity between *I. scapularis* and *I. ricinus* homologs. The accession numbers are shown together with corresponding percent identity for nucleotide (nt) and amino acid (aa) sequences and *E*-values. The selected *I. scapularis* sequences were blasted against the *I. ricinus* database using the Blastp tool from BLAST, and the sequences with the lowest *E*-value were selected. **(D)** Characterization of the effect of rabbit IgG antibodies on IRE/CTVM20 tick cells apoptosis. **(E)** Characterization of the effect of rabbit IgG antibodies on pathogen infection of IRE/CTVM20 tick cells. The percentage of apoptotic cells was determined by flow cytometry after Annexin V-FITC and PI labeling. *A. phagocytophilum* DNA levels were determined by *msp4* real-time PCR normalizing against tick *rpS4*. Control cells were incubated with rabbit pre-immune IgG (negative control, Control -) or rabbit anti-ISE6 IgG (positive control, Control +). Results were presented as average + S.D. normalized Ct-values and compared between each treatment and negative control by Student's *t*-test with unequal variance (*P* ≤ 0.05; *N* = 4). The selected candidate protective antigens are shown with arrows.

By using these criteria, a total of 12 candidate tick protective antigens were initially selected, and 7 of them fulfilled two of the selection criteria (Figure 3A). The recombinant antigens were produced in *E. coli* and used for the preparation of antigen-specific IgG antibodies in immunized rabbits (Figures 4A,B). These IgG antibodies were then used for the incubation with *I. scapularis* ISE6 cells before infection with *A. phagocytophilum* to characterize the effect on cell apoptosis (Figure 5A) and pathogen infection (Figure 5B). The results showed that anti ISCW005600 and AAY66632 IgG significantly increased the percentage of apoptotic cells when compared to negative control cells incubated with pre-immune IgG (Figure 5A). The incubation of ISE6 cells with rabbit IgG against recombinant antigens significantly decreased pathogen infection for 7 antigens when compared to the negative control (Figure 5B). The positive control cells were incubated with rabbit IgG antibodies against total ISE6 tick cells proteins, which significantly increased cell apoptosis but did not affect pathogen infection when compared to the negative control (Figures 5A,B). The anti-ISE6 antibodies did not affect pathogen infection of tick cells, which as previously discussed (Stuen et al., 2015) was due to the presence of not protective dominant antigens in the protein extract used to immunize rabbits for antibody production. Nevertheless, these results showed that incubation of ISE6 tick cells with IgG antibodies against ISCW005600 and AAY66632 antigens affected both cell apoptosis and pathogen infection, and were therefore selected as the candidate tick protective antigens for further characterization (Figures 5A,B).

### Characterization of selected candidate tick protective antigens

The first step in the characterization of selected candidate tick protective antigens was the identification of *I. ricinus* homologs to evaluate their protective potential in both major tick vector species for *A. phagocytophilum*. The *I. ricinus* homologs for *I. scapularis* ISCW005600 and AAY66632 antigens corresponded to putative salivary gland secreted proteins lipocalins (Beaufays et al., 2008; Schwarz et al., 2013; Valdés et al., 2016) and a lectin pathway inhibitor (Ribeiro et al., 2006; Schuijt et al., 2011), respectively (Figure 5C). At the amino acid level, over 70% sequence identity was obtained for both antigens (Figure 5C), suggesting that these proteins are highly conserved in *I. scapularis* and *I. ricinus*, and may be protective in vaccine preparations against both tick vector species.

Experiments were then conducted to characterize the effect of rabbit IgG antibodies against ISCW005600 and AAY66632 antigens in heterologous *I. ricinus* IRE/CTVM20 cells as described before in the homologous *I. scapularis* ISE6 cells (Figures 5D,E). As in ISE6 tick cells, the results showed that incubation of IRE/CTVM20 tick cells with IgG antibodies against ISCW005600 and AAY66632 antigens affected both cell apoptosis (Figure 5D) and pathogen infection (Figure 5E), supporting the putative effect of vaccination with these antigens in both tick vector species.

**Figure 6:**
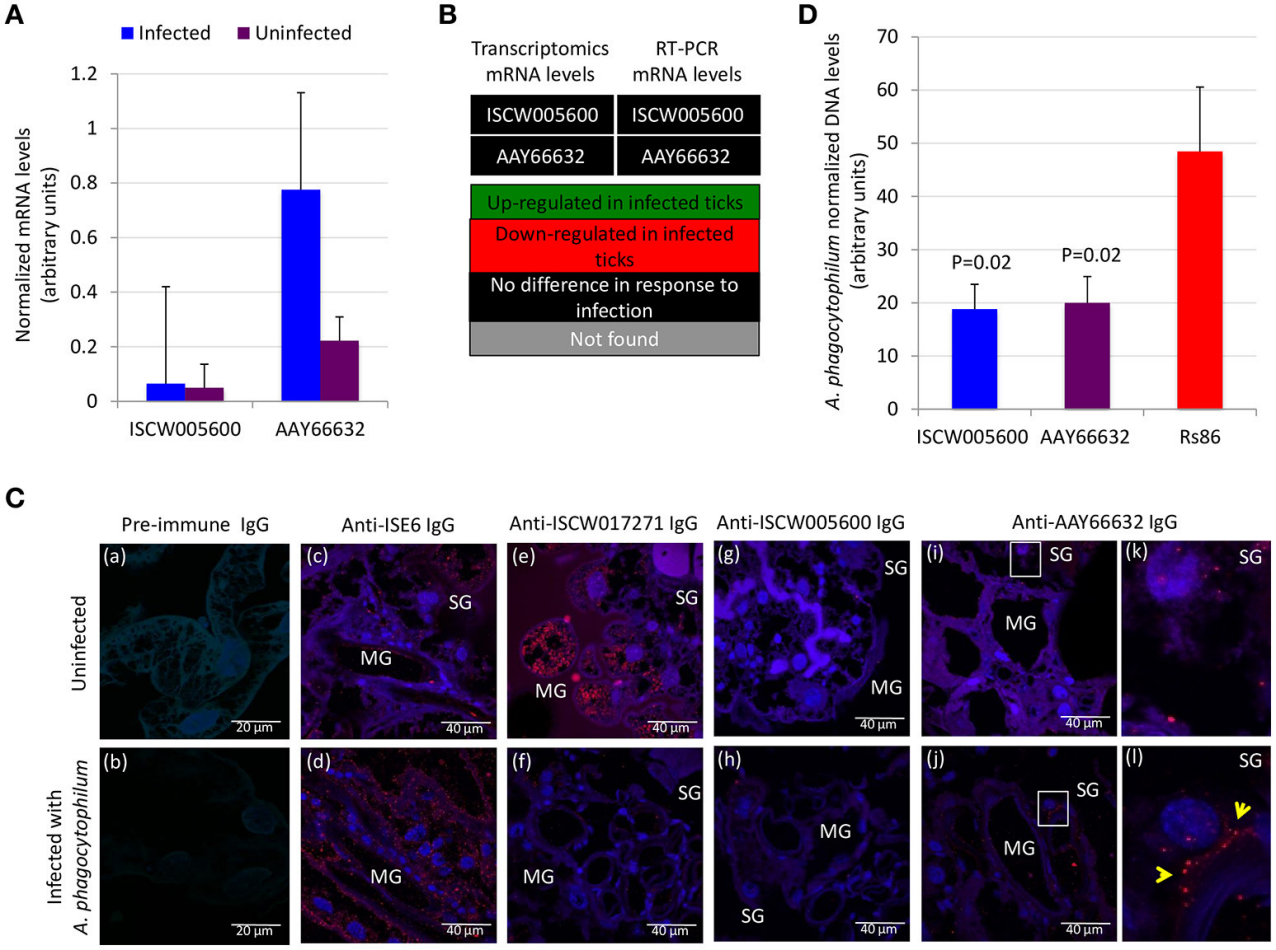
Functional characterization of selected candidate tick protective antigens. **(A)** Results of the RT-PCR analysis of the expression of ISCW005600 and AAY66632 genes in uninfected and *A. phagocytophilum*-infected ISE6 tick cells. Results were presented as average + S.D. normalized Ct-values and compared between infected and uninfected cells by Student's *t*-test with unequal variance (*P* ≤ 0.05; *N* = 4). **(B)** Comparison of the transcriptomics and RT-PCR results for mRNA levels of ISCW005600 and AAY66632 genes in ISE6 tick cells in response to *A. phagocytophilum* infection. Transcriptomics results were obtained from Villar et al. (2015a). **(C)** Representative images of imunofluorescence analysis of uninfected **(a,c,e,g,i,k)** and *A. phagocytophilum*-infected **(b,d,f,h,j,l)** adult female *I. scapularis* midguts (MG) and salivary glands (SG). Tick tissues were stained with rabbit pre-immune control IgG **(a,b)**, anti-ISE6 tick cells IgG **(c,d)**, or anti-tick antigens IgG **(e–l)** labeled with RFP (red) and DAPI (blue). Yellow arrows illustrate a positive staining for AAY66632 in the SG sections in white squares in infected **(l)** but not uninfected **(k)** ticks. **(D)** The *A. phagocytophilum* DNA levels were determined after RNAi in infected ISE6 tick cells treated with ISCW005600 and AAY66632 dsRNAs or control *Rs86* dsRNA. *A. phagocytophilum* DNA levels were determined by *msp4* real-time PCR normalizing against tick *rpS4*. Results are shown as average + S.D. normalized Ct-values and compared between treated and control groups by Student's *t*-test with unequal variance (*P* < 0.05; *N* = 5 biological replicates).

Functional analyses were conducted to gain additional insight into the possible protective mechanisms for these antigens. The expression of ISCW005600 and AAY66632 was determined by RT-PCR and did not change in response to *A. phagocytophilum* infection of ISE6 tick cells (Figure 6A), a result that agreed with previous results of transcriptomics analysis (Villar et al., 2015a; Figure 6B). The IFA in uninfected and *A. phagocytophilum*-infected *I. scapularis* females showed that as expected, a negative and positive staining was obtained with pre-immune and anti-ISE6 IgG in infected ticks, respectively (Figures 6Ca–d). The ISCW017271 antigen, which protein levels were highly under-represented in response to infection in both midguts and salivary glands (Figure 3A), was used to validate proteomics results. The IFA using anti-ISCW017271 IgG antibodies showed a positive staining in uninfected (Figure 6Ce) but not infected cells (Figure 6Cf), thus corroborating the proteomics results. For the selected candidate tick protective antigens, the IFA with anti-ISCW005600 IgG did not produce any positive staining (Figures 6Cg–h), in accordance with proteomics results (Figure 3A). However, for the AAY66632 antigen, a positive staining was obtained in salivary glands from infected ticks after IFA with anti-AAY66632 antibodies (Figures 6Ci,j). The positive staining in infected (Figure 6Cl) but not uninfected (Figure 6Ck) ticks formed a membrane-like structure in salivary glands (arrows in Figure 6Cl), and also corroborated the proteomics results for this antigen (Figure 3A).

Gene knockdown by RNAi in ISE6 tick cells resulted in significantly lower *A. phagocytophilum* infection levels for both antigens when compared to control cells using the unrelated Rs86 dsRNA (Figure 6D). These results suggested that although ISCW005600 and AAY66632 mRNA levels did not change in response to infection of ISE6 tick cells, which constitute a model for tick hemocytes involved in pathogen infection and immune response (Villar et al., 2015a; Alberdi et al., 2016), they may play a role in *A. phagocytophilum* infection.

These results encouraged a final experiment to evaluate the potential effect of ISCW005600 and AAY66632 as vaccination antigens to reduce tick infestations and reproduction. An artificial tick feeding system using silicone membranes was used in this experiment (Kröber and Guerin, 2007; Krull et al., 2017). Although the development of standardized *in vitro* feeding methods for ixodid ticks has been hampered by their complex feeding behavior and the long duration of their blood meal, recent developments provide a valuable tool for the study of tick physiology, tick-host-pathogen interactions and the discovery of drugs and other control interventions without the use of experimental animals (Kröber and Guerin, 2007; Bonnet and Liu, 2012; Sojka et al., 2015; Tajeri et al., 2016; Krull et al., 2017; Trentelman et al., 2017). *I. ricinus* ticks were selected for artificial feeding and the results shown here supported an effect of antibodies against *I. scapularis* antigens (Figure 4B) on *I. ricinus* ticks (Figures 5D,E).

**Figure 7:**
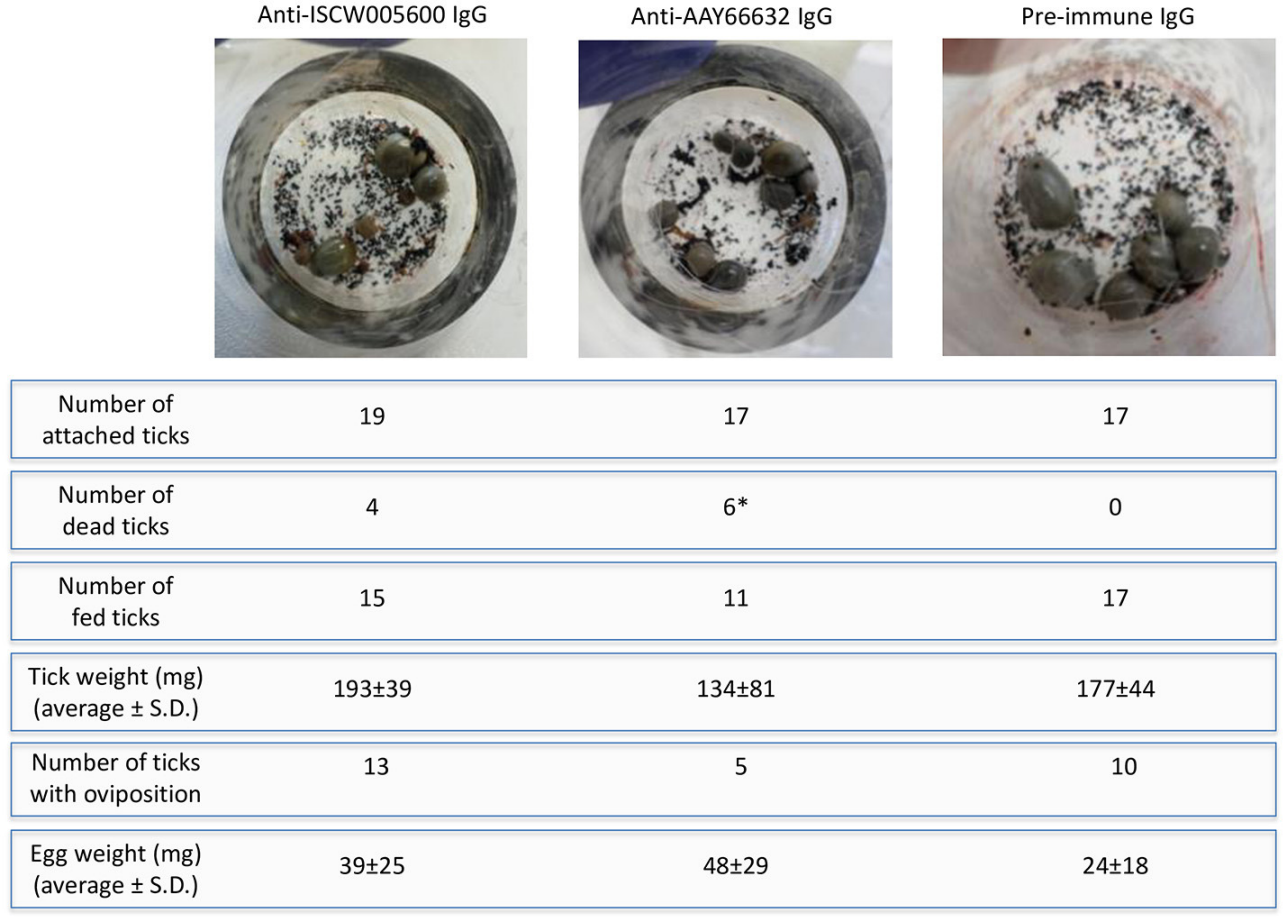
Artificial tick feeding. Artificial tick feeding was conducted as previously described (Krull et al., 2017) with 17–19 female and 3 male *I. ricinus* ticks per feeding unit. Environmental conditions were set at 20°C, 80% relative humidity, 5% CO_2_ and 15 h light/9 h dark. Feeding units containing partially engorged ticks were placed on a six well plate and ticks were fed for 36 h with blood supplemented with 1 mg/ml of pre-immune or antigen-specific purified IgG. Dead and detached engorged ticks were removed, and engorged females that detached were weighed and stored individually in 2 ml Eppendorf tubes with pierced lids, which were kept in desiccators with approximately 90% relative humidity at RT. Ticks were assessed for egg mass 8 weeks post-feeding. The number of dead/fed ticks and thus with oviposition were compared between groups by a Fisher's exact test (^*^P = 0.02).

On the artificial feeding device, the number of attached ticks was similar between groups, but the number of dead ticks increased after feeding on anti-antigen IgG and was significantly higher in ticks fed on anti-AAY66632 antibodies when compared to control ticks fed on pre-immune IgG (Figure 7). Significant differences were not observed between groups in tick weight, number of ticks with oviposition and egg weight, but a tendency in the reduction in the number of ticks with oviposition was also observed in ticks fed on anti-AAY66632 IgG (Figure 7). Although the number of ticks used for artificial feeding was limited due to experimental conditions, the results suggested an effect of anti-ISCW005600 and anti-AYY66632 antibodies on tick mortality and a reduction in the number of ticks with oviposition for anti-AYY66632 antibodies.

These results suggested that the selected candidate tick protective antigens might constitute effective vaccine antigens to control tick vector infestations and prevent or control pathogen infection, and therefore could be selected for future vaccination trials.

### Putative mechanisms of protection for vaccines based on selected candidate tick protective antigens

After the successful completion of the main objective of this study, which was the identification of tick candidate tick protective antigens for the control of vector infestations and *A. phagocytophilum* infection, a question arose about the putative protective mechanisms of the selected candidate protective antigens. The answer to this question may assist in the selection of additional candidate protective antigens following the vaccinomics pipeline (Figure 1), and the evaluation of possible combinations of antigens with different functions to enhance vaccine efficacy (de la Fuente and Merino, 2013).

Both selected candidate tick protective antigens were grouped into the evasion of host defense response BP (Figure 3B). The ISCW005600 secreted histamine binding protein appears to be a salivary lipocalin (Beaufays et al., 2008; Schwarz et al., 2013). Lipocalins are a family of salivary gland secreted proteins that play a role in evasion of host immune and inflammatory responses by competing for histamine or serotonin binding (Paesen et al., 2000; Mans, 2005; Beaufays et al., 2008; Valdés, 2014; Valdés et al., 2016). Therefore, these proteins play an important role during tick feeding. The genes encoding for these proteins are up-regulated during tick feeding (Kim et al., 2016; Valdés et al., 2016; Ribeiro et al., 2017) and pathogen infection (Ayllón et al., 2015; Valdés et al., 2016). Additionally, lipocalins were also produced in tick midguts and up-regulated in response to *A. phagocytophilum* infection (Ayllón et al., 2015; Figure 3A), suggesting as reported in other organisms (Cassidy and Martineau, 2014; Abella et al., 2015) a role for these proteins in tick innate immune response to infection. Therefore, lipocalins may have a dual role in tick-pathogen interactions. These proteins may facilitate pathogen transmission by reducing host inflammatory responses (Valdés et al., 2016), but control tick infection by depleting strategic compounds for pathogens (Ferreira et al., 2015). In humans, lipocalins have also been shown to regulate apoptosis by inducing or inhibiting this process under different physiological conditions (Chakraborty et al., 2012; Abella et al., 2015). Based on the results obtained here with anti-ISCW005600 antibodies and RNAi (Figures 5A,B,D,E, 6D, 7), ISCW005600 may function to inhibit tick cell apoptosis and facilitate *A. phagocytophilum* infection with a possible role during tick feeding (Figure 7). Therefore, the proposed protective mechanisms for vaccines containing this antigen may include reduction of tick infestations by increasing cell apoptosis and reducing protective capacity to host response while reducing pathogen infection and transmission. Tick lipocalins have been proposed before as vaccine antigens for the control of tick infestations (de Castro et al., 2016; Manzano-Román et al., 2016), but only low partial protection have been reported in soft ticks, *Ornithodoros moubata* fed on immunized rabbits (Manzano-Román et al., 2016).

The AAY66632 antigen is a secreted lectin pathway inhibitor (Ribeiro et al., 2006; Schuijt et al., 2011), which is involved in the inhibition of the innate immune response complement lectin pathway (CLP). The CLP is involved in host response to infection with different pathogens (Evans-Osses et al., 2013). The CLP is activated when mannan-binding lectins or ficolins bind to patterns of carbohydrates or acetyl groups on the surface of protozoan, virus, fungi, or bacteria (Runza et al., 2008; Héja et al., 2012; Evans-Osses et al., 2013). In ticks, the inhibition of the complement system during and after blood feeding is critical for tick feeding success and development by minimizing damage to the intestinal epithelium as well as avoiding inflammation and opsonization of salivary molecules at the bite site (Wikel and Allen, 1977; Franco et al., 2016). Therefore, complement inhibitors are present in both tick saliva and midgut (Barros et al., 2009; Mendes-Sousa et al., 2013; Ayllón et al., 2015) (Figure 3A). The presence and activity of salivary anti-complement molecules has been well characterized in *Ixodes* spp. ticks including the *A. phagocytophilum* vectors, *I. scapularis* (Valenzuela et al., 2000; Tyson et al., 2007, 2008, Schuijt et al., 2011) and *I. ricinus* (Lawrie et al., 1999, 2005, Daix et al., 2007; Couvreur et al., 2008). Moreover, tick lectin pathway inhibitors have been shown to facilitate *Borrelia burgdorferi* pathogen infection and transmission (Schuijt et al., 2011; Wagemakers et al., 2016). Our results supported a role for AAY66632 in tick feeding success (Figure 7), the inhibition of tick cell apoptosis (Figures 5A,D) and facilitation of *A. phagocytophilum* infection (Figures 5B,E, 6D). Therefore, the proposed protective mechanisms for vaccines based on this antigen may include reduction of tick infestations by affecting tick attachment and/or feeding, while reducing pathogen infection and transmission. The protective capacity of vaccines containing this antigen has not been reported.

## Conclusions

The main objective of this study was to apply a vaccinomics approach to the identification and characterization of candidate tick protective antigens for the control of vector infestations and *A. phagocytophilum* infection. The vaccinomics pipeline developed in this study was applied to tick-*A. phagocytophilum* interactions and resulted in the identification of two candidate tick protective antigens that could be selected for future vaccination trials. The results showed that *I. scapularis* ISCW005600 and AAY66632 and *I. ricinus* homologs constitute candidate protective antigens for the control of vector infestations and *A. phagocytophilum* infection. Both lipocalin (ISCW005600) and lectin pathway inhibitor (AAY66632) are involved in the tick evasion of host defense response and pathogen infection and transmission, but targeting different immune response pathways. Therefore, based on the putative function of these antigens, vaccine protective mechanisms were proposed that supported antigen combination to improve vaccine efficacy. The vaccinomics pipeline proposed here could be used to continue the identification and characterization of candidate tick protective antigens for the development of effective vaccines for the prevention and control of HGA, TBF, and other forms of anaplasmosis caused by *A. phagocytophilum*.

## Author contributions

JD conceived the study. MC, PA, IF, MV, CK, and AN performed the experiments. MC, PA, MV, CK, AN, and JD performed data analyses. JD, MC, and PA wrote the paper, and other coauthors made additional suggestions and approved the manuscript.

### Conflict of interest statement

The authors declare that the research was conducted in the absence of any commercial or financial relationships that could be construed as a potential conflict of interest.
